# Feeble-Mindedness; Its Cause and Consequences

**Published:** 1915-06

**Authors:** Malone Duggan


### Book Reviews.

We are in receipt of a small book on the Federal Narcotic Law, which has been prepared by Alberdean Currier of the Chicago bar and Taylor R. Forbes, late with the United States Board of Food and Drug Inspection. It is the aim of the book to present a concise, accurate analysis of the law and regulations. It is indexed so that the busy practitioner may turn at once to the points upon which he desires information. The booklet is published by the National Association of Retail Druggists, 122 National Avenue, Chicago. Price, 25 cents per copy.

FEEBLE-MINDEDNESS; ITS CAUSE AND CONSEQUENCES. By Henry Herbert Goddard, Ph. D. Macmillan Co., New York, 1914. Price, $4.00.

Confronted with the fact that the number of feeble-minded people in the United States totals more than the combined population of the four largest cities in our State; that one-half of all paupers, habitual drunkards and prostitutes belong to this class; that from one-fourth to one-half of all criminals in prisons are recruited from this population, and that 2 per cent of all school children are morons, a consideration of this subject at once appeals to every man and woman who has the interest of humanity and society at heart.

Dr. Goddard, in this book, gives us what appears to be the most thorough and important work that has ever been done in this field of investigation. A complete family history of the inmates of the Vineland Institution has been compiled, and through the aid of laboratory methods the questions of heredity and management have been painstakingly studied. From his researches, Dr. Goddard deducts the important facts: First, that feeble-mindedness is a heritable unit; second, that normal intelligence is also a heritable unit, and that they both comply with the Mendelian laws of heredity. This confirmation is far-reaching in its effects, and will greatly stimulate the interest in the further application of eugenics. Indeed, when once the remedy is known, the responsibility of applying it increases, and today society cannot shrink from its duty in this urgent matter.

The relation of the feeble-minded, especially the large moron group, to every class of society, is convincingly. shown. Heredity and eugenics are very clearly presented, so that this relationship can be fully appreciated.

Every school community is vitally interested, or should be, in the care and management of the *backward* child, and in determining the class of work suitable to its intelligence.

Crime, pauperism, prostitution all take on a new appearance when discussed from the sense of responsibility. Efficiency and adaptation to environment, practical philanthropy and social service will receive a new impetus from the further understanding of this subject.

The physician can better council his "ne'er do wells," and aid further in social betterment by his knowledge of these facts. In fact, this is a book that is urgently needed by every class of society, minister, physician, social worker, judge on the bench, lawmaker and the parent.

MALONE DUGGAN, M. D.

